# *Palladin* Mutation Causes Familial Pancreatic Cancer and Suggests a New Cancer Mechanism

**DOI:** 10.1371/journal.pmed.0030516

**Published:** 2006-12-12

**Authors:** Kay L Pogue-Geile, Ru Chen, Mary P Bronner, Tatjana Crnogorac-Jurcevic, Kara White Moyes, Sally Dowen, Carol A Otey, David A Crispin, Ryan D George, David C Whitcomb, Teresa A Brentnall

**Affiliations:** 1 Department of Medicine, University of Pittsburgh, Pittsburgh, Pennsylvania, United States of America; 2 Department of Medicine, University of Washington, Seattle, Washington, United States of America; 3 Department of Pathology, Cleveland Clinic Foundation, Cleveland, Ohio, United States of America; 4 Molecular Oncology Unit, Cancer Research United Kingdom, Barts and the London School of Medicine and Dentistry, London, United Kingdom; 5 Department of Cell and Molecular Physiology, University of North Carolina at Chapel Hill, North Carolina, United States of America; 6 Department of Cell Biology and Physiology, University of Pittsburgh, Pittsburgh, Pennsylvania, United States of America; 7 Department of Human Genetics, University of Pittsburgh, Pittsburgh, Pennsylvania, United States of America; Cambridge Research Institute/Cancer Research UK, United Kingdom

## Abstract

**Background:**

Pancreatic cancer is a deadly disease. Discovery of the mutated genes that cause the inherited form(s) of the disease may shed light on the mechanism(s) of oncogenesis. Previously we isolated a susceptibility locus for familial pancreatic cancer to chromosome location 4q32–34. In this study, our goal was to discover the identity of the familial pancreatic cancer gene on 4q32 and determine the function of that gene.

**Methods and Findings:**

A customized microarray of the candidate chromosomal region affecting pancreatic cancer susceptibility revealed the greatest expression change in *palladin (PALLD),* a gene that encodes a component of the cytoskeleton that controls cell shape and motility. A mutation causing a proline (hydrophobic) to serine (hydrophilic) amino acid change (P239S) in a highly conserved region tracked with all affected family members and was absent in the non-affected members. The mutational change is not a known single nucleotide polymorphism. *Palladin* RNA, measured by quantitative RT-PCR, was overexpressed in the tissues from precancerous dysplasia and pancreatic adenocarcinoma in both familial and sporadic disease. Transfection of wild-type and P239S mutant *palladin* gene constructs into HeLa cells revealed a clear phenotypic effect: cells expressing P239S palladin exhibited cytoskeletal changes, abnormal actin bundle assembly, and an increased ability to migrate.

**Conclusions:**

These observations suggest that the presence of an abnormal *palladin* gene in familial pancreatic cancer and the overexpression of palladin protein in sporadic pancreatic cancer cause cytoskeletal changes in pancreatic cancer and may be responsible for or contribute to the tumor's strong invasive and migratory abilities.

## Abstract 2

The presence of abnormal*palladin* in familial pancreatic cancer and its overexpression in sporadic pancreatic cancer leads to cytoskeletal changes and may be responsible for the tumor's invasive and migratory abilities.

## Editors' Summary

**Background.:**

Pancreatic cancer is a leading cause of cancer-related death in the US. Because it causes few symptoms in its early stages, pancreatic cancer is rarely detected until it has spread (metastasized) around the body. Pancreatic tumors can occasionally be removed surgically but the usual treatment is radio- or chemotherapy, and neither of these is curative; most patients die within a year of diagnosis. As in other cancers, the cells in pancreatic tumors have acquired genetic changes (mutations) that allow them to divide uncontrollably (normal cells divide only to repair damaged tissue). Other mutations alter the shape of the cells and allow them to migrate into (invade) other areas of the body. These mutations usually arise randomly—the cells in the human body are bombarded by chemicals and other agents that can damage their DNA—and cause “sporadic” pancreatic cancer. But some people inherit mutated genes that increase their susceptibility to pancreatic cancer. These people are recognizable because pancreatic cancer is more common in their families than in the general population.

**Why Was This Study Done?:**

The identification of the genes that are mutated in familial pancreatic cancer might provide insights into how both inherited and sporadic cancer develops in the pancreas. Such information could suggest ways to detect pancreatic cancers earlier than is currently possible and could identify new therapeutic targets for this deadly disease. Previous work by the researchers who did this study localized a gene responsible for inherited pancreatic cancer to a small region of Chromosome 4 in a family in which pancreatic cancer is very common (Family X). In this study, the researchers identified which of the genes in this region is likely to be responsible for the susceptibility to pancreatic cancer of Family X.

**What Did the Researchers Do and Find?:**

The researchers made a DNA microarray (a small chip spotted with DNA sequences) of the 243 genes in the chromosomal region linked to pancreatic cancer in Family X. They used this to examine gene expression in dysplastic pancreatic tissue from a Family X member (pancreatic dysplasia is a precancerous lesion that precedes cancer), in normal pancreatic tissue, and in samples from sporadic pancreatic cancers. The most highly overexpressed (compared to normal tissue) gene in both the Family X tissue and the sporadic cancers encoded a protein called palladin. Palladin is a component of the cytoskeleton (a structure that helps to control cell shape and motility) and it organizes other cytoskeletal components. Next, the researchers quantified the expression of *palladin* RNA in an independent set of normal and cancerous pancreatic samples, and in precancerous pancreatic tissue taken from Family X members and from people who inherit pancreatic cancer but who were not in Family X. This analysis indicated that *palladin* was overexpressed early in sporadic and inherited pancreatic cancer development. Sequencing of the *palladin* gene then uncovered a mutation in *palladin* that was present in Family X members with pancreatic cancer or precancerous lesions but not in unaffected members. This specific mutation, which probably affects palladin's interaction with another cytoskeletal protein called alpha-actinin, was not found in sporadic cancers although many sporadic cancer cell lines had abnormal expression of alpha-actinin protein in addition to palladin protein. Finally, the researchers showed that the introduction of mutated palladin into a human cell line growing in the laboratory increased its migration rate and disrupted its cytoskeleton.

**What Do These Findings Mean?:**

These results strongly suggest that mutated *palladin* is involved in the development of familial pancreatic cancer. Because genes tend to be inherited in groups, there is still chance that a mutation in a nearby gene could be responsible for the increased susceptibility to pancreatic cancer in Family X. However, the data showing *palladin* overexpression in sporadic tumors and alterations of cell behavior in the laboratory after introduction of the mutated gene make this unlikely. To prove the involvement of *palladin* in pancreatic cancer, *palladin* mutations must now be identified in other familial cases and the overexpression of palladin in sporadic cancers must be explained. The results here nevertheless provide an intriguing glimpse into a potential new mechanism for cancer development in the pancreas and possibly other tissues, one in which abnormalities in palladin function or expression (or in the proteins with which it associates) drive some of the changes in cell migration, shape, and size that characterize cancer cells.

**Additional Information.:**

Please access these Web sites via the online version of this summary at http://dx.doi.org/10.1371/journal.pmed.0030516

- US National Cancer Institute, information on pancreatic cancer for patients and health professionals
- MedlinePlus encyclopedia entry on pancreatic carcinoma
- Cancer Research UK, information for patients about pancreatic cancer
- Johns Hopkins University, information on pancreatic cancer that includes details on familial cancer
- CancerQuest, information provided by Emory University about how cancer develops

## Introduction

Pancreatic adenocarcinoma is the fourth leading cause of cancer death in the United States, and the third leading cause in individuals aged 40–59 years [1]. Pancreatic cancer is difficult to detect, resistant to treatment, and is usually discovered after it has metastasized. Nearly every person who develops pancreatic cancer will die from it, the majority within the first year of diagnosis [2]. The identification of high-risk individuals and elucidation of key oncogenic pathways are therefore priorities for scientists and physicians hoping to change this cancer's dismal prognosis.

Familial clustering of pancreatic cancers is commonly recognized, occurring in at least 10% of all pancreatic cancer [3,4]. The risk of pancreatic cancer increases with each family member who is affected [5,6]. Growing evidence suggests that some apparently sporadic pancreatic cancers are actually caused by inherited genetic susceptibility [4]. Pancreatic cancer can also be inherited as part of a familial cancer syndrome such as that associated with *BRCA2* mutations, Peutz-Jeghers syndrome, and familial atypical mole and melanoma syndrome [4]. In those cases, the family pedigree shows evidence of other cancers such as breast or intestinal tumors, or melanomas, in addition to pancreatic tumors. The vast majority of familial pancreatic cancer is not accounted for by such known cancer syndromes. Discovery of a gene (or genes), that when mutated in the germline cause heritable pancreatic adenocarcinoma, could provide important insights into the biology of the pancreatic cancers, and potentially of non-pancreatic cancers as well. Such discoveries have been difficult because the rapid demise of pancreatic cancer patients hampers collection of biological samples for genotyping.

We previously reported an exceptional family, Family X, in which pancreatic adenocarcinoma is inherited in an autosomal dominant fashion with high penetrance [7]. In order to identify living carrier family members prior to the onset of cancer, we developed an endoscopic surveillance program that assists in the detection of pancreatic precancerous dysplasia [8,9]. This condition, also called pancreatic intraepithelial neoplasia (PanIN), is the precancerous lesion of both sporadic and familial pancreatic cancers. PanIN is graded from 1 to 3 with increasing neoplastic progression: hyperplasia (PanIN 1) to low-grade dysplasia (PanIN 2) to carcinoma in situ (PanIN 3). PanIN lesions of all three grades are prominent throughout the pancreatic tissue of affected Family X members prior to cancer formation [10]. Through our pancreatic cancer surveillance program we were able to identify the members of Family X who had impending cancer formation (histologic conformation of PanIN 2 and 3 in the pancreas). This program allowed us to identify the patients at a curable stage of their disease and to genotype them.

Four generations of Family X include 18 cases of either adenocarcinoma (*n* = 9) or histologically proven precancerous PanIN 2 and 3 (*n* = 9) (Figure 1). Through genotyping of 35 family members, a pancreatic adenocarcinoma susceptibility locus was previously mapped to the chromosomal location 4q32–34 [11]. The work to identify the gene of interest in this region has been a considerable challenge because of the size (16 Mb) and the number of genes (approximately 250) localized to this region [12]. We now report the identification of this familial pancreatic cancer gene and the assessment of that gene's involvement in familial and sporadic pancreatic cancer.

**Figure 1:**
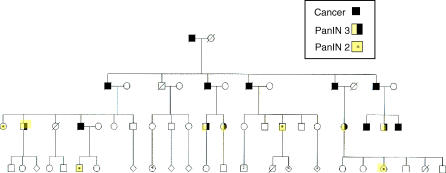
Family X Pedigree Nine members of this family were diagnosed with pancreatic cancer and nine with pancreatic precancer (five with carcinoma in situ [PanIN 3] and four with low-grade dysplasia [PanIN 2]).

## Methods

### Biological Samples

All human samples (blood, pancreas, and constitutional tissues such as duodenum and lymph nodes) were collected and used under protocols approved by the Institutional Review Board at the University of Washington (Seattle, Washington, United States) and the University of Pittsburgh.

Family X is of western European descent. In this kindred, pancreatic adenocarcinoma is inherited in an autosomal dominant fashion with high penetrance [7]. All study participants from Family X or other familial pancreatic cancer kindreds were ascertained at the University of Washington, with additional samples from patients without cancer (controls) or with pancreatic adenocarcinoma from all participating institutions.

Additional normal pancreas samples were obtained commercially from Stratagene (La Jolla, California, United States), Clontech (Mountain View, California, United States), Biochain (Hayward, California, United States), Chemicon (Temecula, California, United States), and Ambion (Austin, Texas, United States). Surgically resected pancreatic tissue samples were snap-frozen in liquid nitrogen or on dry ice immediately after gross identification and approval by the clinical pathologists responsible for the cases. Adjacent and representative frozen sections and fixed sections were analyzed and pathological diagnosis made by a qualified pathologist (MPB). Tissue samples remained frozen at −80 °C until use. In addition, pancreatic cancer cell lines (FA6, HPAF, PANC 1, HS766T, IMMPC2, SUIT 2, and PATU2) were obtained courtesy of Dr. Nicholas Lemoine (Molecular Oncology Unit, Cancer Research United Kingdom, Barts, and the London School of Medicine and Dentistry, London, United Kingdom) and author TCJ. The human pancreatic ductal epithelium (HPDE) cell line was obtained courtesy of Dr. Ming-Sound Tsao (Department of Medical Biophysics, University of Toronto, Toronto, Ontario, Canada).

### Custom Microarrays

Unigenes represent a nonredundant set of gene-oriented clusters of cDNA clones assigned to a unique gene and/or genomic location. Unigenes for this study were identified using the UCSC genome maps (draft sequence 12/01) (http://genome.ucsc.edu/cgi-bin/hgGateway/) and NCBI maps. We identified 243 Unigene clones mapping between markers D4S413 and D4S299. Seventeen clones were included as housekeeping genes and 25 additional clones were included because of their location in a region of the genome that is frequently lost in pancreatic cancer. Clones were acquired from Research Genetics (now Invitrogen, Carlsbad, California, United States), from RZPD (German Resource Center for Genome Research, Berlin, Germany), and from the University of Pittsburgh Genomics and Proteomics Core Laboratories (Pittsburgh, Pennsylvania, United States). Identification of all clones and accurate locations on chromosome 4q were confirmed by DNA sequence. Clones were amplified, purified, and arrayed onto glass slides in sextuplicates as previously described [13].

### Hybridization

Slides were scanned with a GMS 418 scanner (Genetic MicroSystems brand, Affymetrix, Santa Clara, California, United States). The Cy-5 and Cy-3 images were overlaid, and raw data were generated for both channels using the ImaGene program United (Bioinformatics, Calgary, Alberta, Canada). Microarray analysis was performed using the Gene Expression Differential Analysis tool (caGEDA), a web application specifically developed for cancer microarray data analysis (http://bioinformatics.upmc.edu/GE2/GEDA.html) [14].

### Quantitative RT-PCR

Quantitative RT-PCR (qRT-PCR) was carried out using the 5′ nuclease assay and an Applied Biosystems 7700 Sequence Detection Instrument (with TaqMan) (http://www.appliedbiosystems.com). Expression analysis utilized whole tissues. *Palladin (PALLD)* expression was measured relative to the endogenous control, *GAPDH,* using the comparative C_T_ method described previously [15]. The cDNAs were generated at two different RNA input concentrations (10 ng/μl and 4 ng/μl) and TaqMan reactions with the endogenous control were run in duplicate from both RT reactions. *Palladin* TaqMan reactions were performed in triplicate using RT reactions with 10 ng/μl reactions. A calibrator RNA composed of 50 ng/μl universal reference RNA (Stratagene; #40000–41) and 50 ng/μl colon RNA (Ambion; #7986) was included on every amplification plate to allow for the comparison of samples run at different times. RT-negative controls were run on each plate to ensure that no amplification occurred in the absence of cDNA. Primers were purchased from Applied Biosystems (Hs99999905_m1 for *GAPDH* and Hs00363100_m1 for *palladin*).

### Statistical Analysis

We used the J5 test and normalized the data with log2 and Z transformation as previously described [14]. The J5 test compares the difference in mean expression for a given gene to the magnitude (absolute value) of mean difference in all of the genes on an array. To evaluate group differences on qRT-PCR statistically, an overall one-way, 4-group analysis of variance was performed. Pairwise group comparisons were analyzed using Fisher's least squares difference test with a significance level of *p* < 0.05.

### DNA Sequencing of Candidate Genes

While development of the custom microarray was underway, we identified 22 potential candidate genes in the 4q32–34 region and prioritized them according to the likelihood that they play a role in pancreatic neoplastic transformation. Twenty of the genes were sequenced in entirety, while the last two genes were partially sequenced because the *palladin* gene mutation had been discovered (Table 1). Primers and PCR conditions for these 22 genes are available upon request. Sequencing was carried out with the Big Dye V3.1 kit (ABI, Foster City, California, United States).

**Table 1:**
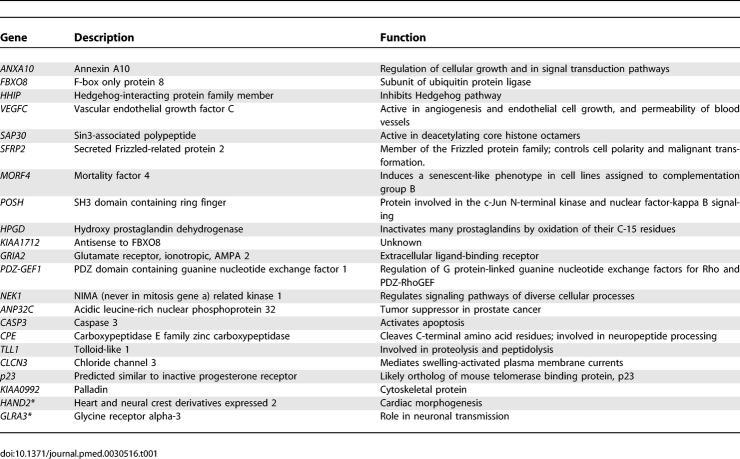
Candidate Genes Sequenced in Region 4q32–34

### Copy Number Assessment of Palladin

We used a custom-manufactured high-density *palladin* array to assess the copy number of *palladin* in dysplastic tissue (NimbleGen Systems, Madison, Wisconsin, United States). The sequence of *palladin* was tiled with oligonucleotides that started every 60 bp. Comparative genomic hybridization was performed using genomic DNA from fresh frozen samples of PanIN 2 tissue and matching lymphocytes from two separate Family X members. Pancreatic ductal epithelial cells were purified from pancreatic tissue samples by using epithelial antibody EpCAM and magnetic beads according to the manufacturer's instructions (CELLection Epithelial Enrich Kit; Dynal, Oslo, Norway). The DNA purified from the samples was hybridized to the high-density *palladin* array by the NimbleGen Service Laboratory. Fluorescence intensity raw data were obtained from scanned images of the oligonucleotide tiling arrays by using Nimblescan 2.0 extraction software and the results were analyzed at the NimbleGen Service Laboratory.

### Protein Analysis by Western Blot

Size fractionation (SDS-PAGE) was performed on 20 μg of protein from each pancreatic cell line sample. The samples were individually loaded onto a gel, separated through electrophoresis, and then blotted onto a nitrocellulose membrane according to manufacturer's protocol (Amersham Biosciences, Piscataway, New Jersey, United States). The polyclonal palladin antibody (ab 621) was created as previously described [16]. A 1:2,000 dilution was used for Western blotting. The ECL plus kit (Amersham Biosciences) was used to detect protein in the Western blot.

### *Palladin* Constructs

The human wild-type (WT) *palladin* construct was made by PCR-cloning the entire coding sequence from a human *palladin* cDNA clone (human cDNA clone hk07554, a gift from Dr. Takahiro Nagase of Kazusa DNA Research Institute) into the sites of EcoRI and BamHI of phrGFP IIN vector (Stratagene), downstream of, and in frame with, the green fluorescent protein (GFP) tag. To create the Family X mutant construct (FX), we used Quick-Change Multi Site-Directed Mutagenesis (Stratagene) with primers centered at the Family X mutation (P239S), according to manufacturer's protocol. Briefly, (1) 100 ng of WT construct was added as a template to a PCR cocktail containing 2.5 μl of 10× mutagenesis buffer, 100 ng of mutagenesis primer containing the Family X mutation, 250 μM each dNTP, 1 μl QuickChange enzyme blend and 1 μl QuickSolution. The PCR cycling parameters were one cycle of: 1 min at 95 °C, followed by 30 cycles of 1 min at 95 °C, and 15 min at 65 °C; (2) the parental template DNA was treated with DpnI (10 U) at 37 °C for 60 min; (3) 4 μl of this reaction was transformed into XL10 Gold Ultracompetent cells (Stratagene); and (4) several clones were chosen for PCR and/sequencing to confirm the incorporation of the mutation.

### HeLa Cell Transfection with Wild-Type and Mutated *Palladin*

Transfection was performed using a Fugene kit (Roche Diagnostics [http://www.rochediagnostics.com]) on human cancer epithelial cell lines (HeLa cells) according to the manufacturer's protocol. Briefly, (1) one day before the transfection, the cells were distributed into a six-well plate so that they would be approximately 70% confluent the next day; (2) 3–6 μl of transfection reagent was mixed with 94–97 μl of DMEM and left for 5 min; (3) then 1 μg of construct (either WT or FX mutant construct) was added to the complex and left for 10 min; (4) the complex was then added to the cells in a dropwise fashion; and (5) the expression of the GFP construct was observed the next day. Fluorescence staining was performed on cells fixed in 3.7% formaldehyde and permeabilized with 0.1% Triton X-100. The cells were then stained with 50 μg/ml of TRITC-phalloidin (Sigma) for 40 min, followed by washing with PBS. Finally, DAPI (Sigma) was added at 10 μg/ml to stain the nucleus, and antifade reagent (Invitrogen) was used to prevent auto-color bleaching. The construct/transfection experiments were run in triplicate and assessed blindly by the senior author.

### Cell Migration Assay

HeLa cells were first transfected with constructs of WT, FX, and empty vector respectively for 24 h, and then sorted for GFP-positive cells. The sorted cells continued to grow for 2 d before they were seeded onto a transwell plate for the migration assay. Migration assays were performed on 24-well Transwell cell culture chambers (Corning Costar Corporation, Cambridge, Massachusetts, United States) fitted with multiporous (8-μm pore size) polycarbonate membranes. The upper chambers of the membrane were coated with fibronectin (10 μg/ml in PBS) 24–48 h before the assay. The upper chamber was then filled with 40,000 cells in suspension with 400 μl of medium (DMEM with 10% FCS), and the lower chamber was filled with 500 μl of the same medium. Plates were placed in a humidified CO_2_ incubator at 37 °C for 17 h. Membrane inserts were then removed, fixed by immersing in ethanol five times with 1 s durations, and stained with 0.5% crystal violet dye (Sigma; #C3886) in 20% methanol for 30 min. After gentle rinsing with water, the nonmigratory cells on the upper surface of the membrane were removed with cotton swabs, leaving only the migratory cells within the membrane. The membrane insert was left to dry overnight and then placed in a 96-well plate. The dye was extracted with 30% acetic acid and the plate was placed on a shaker for 10 min to allow the dye to dissolve completely. The colorimetric absorbance was assessed at 590 nm. A transwell without cells was used as a background control. To correct for the cell numbers seeded in each transwell experiment, we measured the optical density of all the original cells in the transwell (migrated and nonmigrated) for each transfected cell type sample. By comparing the optical density from each sample type, we were able to provide a correction factor for any variation in starting cell number between samples. Each sample was done in triplicate.

## Results

### Microarray Expression Analysis Selects *Palladin* as the Candidate Familial Pancreatic Cancer Gene

Sequencing of candidate genes in the 4q32–34 region was time-consuming and ultimately unsuccessful (see Candidate Gene Sequencing, below). In order to better identify the most promising candidate genes we employed a new strategy. We hypothesized that a mutated gene is best detected in very early precancerous dysplastic tissue, where the mutated gene has initiated neoplastic transformation but before the overarching genetic chaos of cancer has occurred. Theoretically, the abnormal gene expression would become apparent, from a molecular standpoint, as the pancreas began to undergo malignant degeneration. If the gene were a tumor suppressor gene, we hypothesized that expression would be lost; if it were a proto-oncogene, expression would increase. To perform the expression analysis, we narrowed the search to expressed sequence tags that were densely mapped to our region of interest. This approach would allow us to interrogate a large amount of closely spaced DNA with one assay. RNA was purified from whole tissue samples containing PanIN 1 from a Family X member, from two normal pancreas (controls), and from ten sporadic pancreatic cancers. The RNA samples were separately profiled on a custom expression microarray to identify gene expression in the 4q32–34 region. The custom microarray was created using 243 sequence-verified Unigene clones located between markers D4S2976 and D4S415, a region slightly larger than the region identified as the pancreatic cancer susceptibility locus (between markers D4S413 and D4S2991). The Unigene clones were spotted in sextuplicates (6×).

As noted above, a total of 13 RNA samples from pancreatic tissue were profiled (Figure 2A). Comparative expression in Family X and the ten sporadic pancreatic adenocarcinoma samples were rank-ordered by their degree of over- or underexpression. Two *palladin* clones (Unigene [http://www.ncbi.nlm.nih.gov/entrez/query.fcgi?db=unigene] accession number KIAA0992) were the most highly overexpressed Unigenes in the ten sporadic pancreatic cancers, and the same clones were also abnormally expressed in the Family X precancerous sample as well, based on a J5 analysis as previously described (Figure 2B) [14]. *Palladin* clones were also shown to be among the most overexpressed genes when we analyzed *palladin* expression by N-fold analysis in Family X and sporadic pancreatic cancers compared to normal pancreas. The overexpression of *palladin* in sporadic cancer was higher compared to the Family X precancerous tissue.

**Figure 2:**
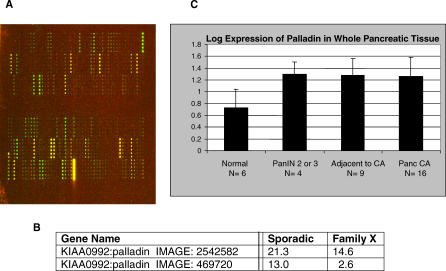
Overexpression of *Palladin* RNA in Sporadic Pancreatic Cancer and in Family X Precancer (A) To narrow down candidate genes we constructed custom 4q32–34 microarrays composed of 243 expressed sequence tags (IMAGE clones), each of which was spotted six times on the array. The microarrays were hybridized with RNA derived from the following target tissues: sporadic pancreatic adenocarcinoma (*n* = 10), Family X pancreatic precancer (*n* = 1), and normal donor pancreas (*n* = 2). Each target tissue was hybridized separately on the arrays and compared to normal pancreas. This array image is representative of one of the sporadic cancer samples versus normal pancreas. Note the consistency of the expression of the six spotted replicates of cDNA clones. Green indicates genes that were underexpressed in cancer compared to normal pancreas; red indicates genes that were overexpressed in cancer compared to normal pancreas, and yellow indicates genes that were expressed in similar levels as normal pancreas. (B) The most overexpressed clones among ten sporadic pancreatic cancers were two different *palladin* clones; both of these clones were significantly overexpressed in Family X precancer. (C) RNA overexpression of *palladin* was confirmed by qRT-PCR. Samples tested were whole pancreatic tissues and included six new normal pancreas samples, four new precancerous tissues (PanIN 2 and 3, or low- and high-grade dysplasia, respectively) from two Family X individuals and two unique familial pancreatic cancer individuals not from Family X, nine histologically normal tissues adjacent to cancer, and 16 new pancreatic cancer tissues. In each case, *palladin* expression was measured relative to the endogenous standardized control, *GAPDH*. Error bars indicate 1 standard deviation above and below the average. *Palladin* RNA is overexpressed early in the process of pancreatic neoplasia, as it is overexpressed in normal-appearing tissue adjacent to sporadic cancer, as well as in precancerous pancreatic tissue. In addition, *palladin* is overexpressed in the precancerous tissue from three different familial pancreatic cancer kindreds (Family X and two other familial pancreatic cancer kindreds).

### *Palladin* RNA is Overexpressed Very Early in the Process of Pancreatic Neoplasia

To validate the findings on the custom microarray, primers directed toward exons 9 and 10 were used to measure expression of the *palladin* gene in whole tissue (Figure 2C). Expression analysis via qRT-PCR was performed in whole pancreas tissue from (1) 16 sporadic pancreatic cancers, (2) four precancerous tissues (two from Family X and two from other familial pancreatic cancer kindreds), (3) nine histologically normal-appearing tissues adjacent to sporadic pancreatic adenocarcinoma (called “normal adjacent”), and (4) six normal pancreas samples. *Palladin* was overexpressed in all of the cancerous and precancerous dysplastic pancreatic tissue, as well as in the histologically normal-appearing tissue adjacent to the cancers when compared to normal pancreas. A significant difference in *palladin* expression levels was detected between normal pancreata and all of the other three groups (cancer, precancer, and normal adjacent): pairwise tests using least significant difference test (*p* < 0.05); one way-four group ANOVA F (3,31) = 5.86, *p* = 0.003. This expression analysis of 35 samples, composed of normal (*n* = 6) and increasingly neoplastic pancreatic tissue (*n* = 29), indicated that *palladin* RNA was overexpressed very early in the development of pancreatic cancer—in normal-appearing whole tissue immediately adjacent to cancer, in the precancer, and in the cancer—of both familial and sporadic forms of the disease.

### Candidate Gene Sequencing Reveals a Mutation in the *Palladin* Gene

While construction of the custom array was taking place, we sequenced some of the candidate genes in the 4q32–34 region selected by their potential role in neoplastic transformation or dysregulation of the pancreas; sequencing of the candidate genes was performed by using constitutional DNA from two affected members of Family X. Twenty genes were fully sequenced and two were partially sequenced (Table 1). When *palladin* was identified by the custom microarray as the most abnormally expressed gene, it was the twentieth and final gene that was fully sequenced. Only *palladin* had a gene mutation; all of the other 19 candidate genes were wild-type.

### Mutation in *Palladin* Tracks Only with the Affected Members in Family X and Causes an Amino Acid Change in the Alpha-Actinin Binding Site

*Palladin* is an extremely large gene spanning 432 Kb, with a total of 31 exons, up to nine probable alternative promoters, and at least eight alternatively spliced transcripts [16–19]. The published literature has described at least three major isoforms of palladin, which have molecular weights 200 kDa, 140 kDa, and 90 kDa. The smallest isoform (90 kDa) is a component of the larger isoforms.

Twenty-eight members from Family X were available for mutational testing of *palladin*: 12 individuals with pancreatic adenocarcinoma (*n* = 3) or histologically verified precancerous dysplasia (*n* = 9) and 16 unaffected individuals. Sequence analysis of *palladin* identified a C to T base pair change at cDNA position 715 in exon 2 that tracks with 100% of the affected family members and none of the unaffected family members*.* The mutation discovered in Family X is contained in the cDNA clone AB023209 (GenBank; http://www.ncbi.nlm.nih.gov/), which encodes a 4,349 nucleotide transcript containing 12 exons and encoding a 772-amino acid protein (90 kDa isoform). The C to T transition causes a proline (hydrophobic) to serine (hydrophilic) change at amino acid 239 in palladin (P239S) (Figure 3). The mutant *palladin* allele is transcribed and expressed at the RNA level in the pancreas from Family X PanIN tissue as documented by qRT-PCR and sequencing.

**Figure 3:**
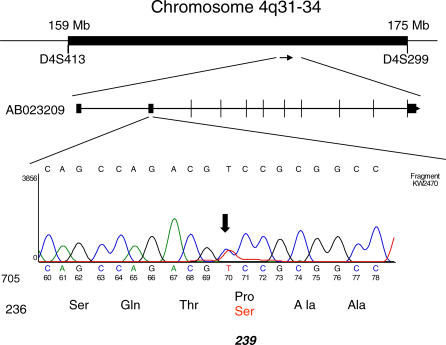
Location and Identification of the Family X Mutation Top black bar indicates the genomic location of the Family X mutation between microsatellite markers D4S413 and D4S299 on Chromosome 4. The center line designates the location of the *palladin* transcript, AB023209, with vertical boxes and lines indicating exons. At the bottom, a small portion of the cDNA sequence shows a C to T base pair change at position 715 (indicated with an arrow), which causes a proline (hydrophobic) to serine (hydrophilic) amino acid change at amino acid 239 (P239S). This location is the essential binding site for alpha-actinin, another key cytoskeletal protein.

The P239S base pair substitution is not a known single nucleotide polymorphism, nor was it detected in the blood of 294 of 295 anonymous controls (the allele frequency was 1 in 590 control alleles). One control blood sample revealed the mutation; unfortunately, the medical history of this individual is unknown. The Family X mutation occurs in a region that is highly conserved across species (Figure 4). This region of *palladin* is likely to be functionally important as it encodes the essential binding site for alpha-actinin, another key cytoskeletal protein (Figure 5) [20].

**Figure 4:**
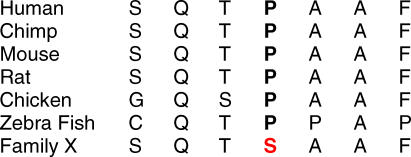
The Binding Site of Alpha-Actinin to Palladin is Highly Conserved across Species The mutation in Family X causes a proline (hydrophobic) to serine (hydrophilic) change in the human amino acid sequence.

**Figure 5:**
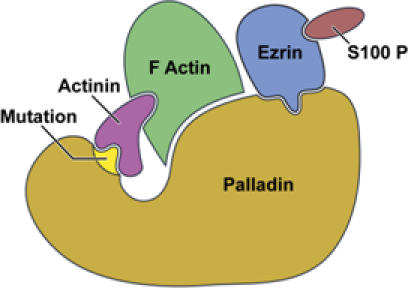
Palladin Is an Alpha-Actinin Binding Protein that Controls Cytoskeletal Formation and Cell Movement Palladin binds other key proteins such as Ezrin [16]. Previous studies have shown that Ezrin and S100P are proteins that are abnormally regulated in pancreatic cancer [35,36].

### *Palladin* Appears to Be a Proto-Oncogene

Loss-of-heterozygosity studies have shown that regions in 4q can be lost in many different cancers, including adenocarcinomas of the pancreas [21–24]. However, comparative genomic hybridization studies have not detected any losses or gains in the 4q32–34 locus in pancreatic cancer [22]. The custom 4q32–34 microarray analysis we performed suggested that *palladin* is not likely to be a tumor suppressor gene (results would reveal loss of expression), but perhaps a proto-oncogene (results would likely reveal overexpression). To validate this conclusion, we determined the number of *palladin* alleles that were present in sorted precancerous epithelial tissue from Family X members; if *palladin* were a tumor suppressor gene one would anticipate allelic loss. A custom-made copy number chip (Nimblegen) was used to measure the number of alleles. The entire *palladin* copy number chip was interrogated using epithelial-sorted dysplastic pancreas samples from two separate Family X members. The copy number of the *palladin* gene was two in every instance. These findings, coupled with the RNA overexpression data, suggest that mutated *palladin* may act as a proto-oncogene.

### Ductal Epithelial Cells Increasingly Overexpressed *Palladin* during Neoplastic Progression

Pancreatic adenocarcinoma encompasses a mixture of tumor cells and a variety of other cell types, especially the prominent desmoplastic cells common to pancreatic adenocarcinomas. To determine whether the *palladin* gene is expressed in the pancreatic ductal cells, we examined the RNA expression in primary HPDE cells with qRT-PCR. Little to no RNA expression was detected in cells from a normal pancreatic ductal epithelium; in contrast, increasing expression was seen in epithelial cells derived from two different affected Family X individuals who had separate tissue samples containing precancer (PanIN 1 [hyperplasia] and PanIN 3 [carcinoma in situ]). The greatest overexpression was seen in ductal epithelial cells taken from a case of sporadic pancreatic adenocarcinoma. The expression of *palladin* increased in primary cultured epithelial cells that were derived from increasingly advanced neoplasia (PanIN 1 [hyperplasia] < PanIN 3 [carcinoma in situ] < cancer) (Figure 6). The tight correlation between *palladin* expression and the neoplastic phenotype present in the ductal epithelial cells (but not in whole pancreatic tissue) is reminiscent of a dose-response curve.

**Figure 6:**
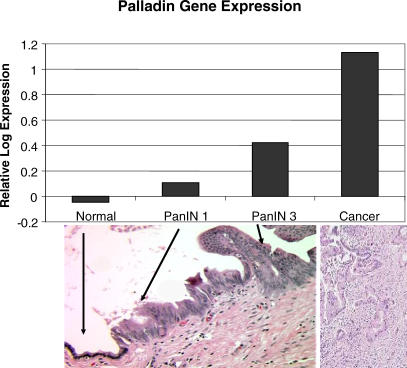
*Palladin* Expression in Primary Cultures of Pancreatic Ductal Cells Derived from Increasingly Neoplastic Samples Expression of *Palladin* RNA increases relative to the degree of precancer to cancer: normal to PanIN 1 (hyperplasia) to PanIN 3 (carcinoma in situ) to cancer. Each bar represents epithelial cell cultures from one person. The PanIN 1 and PanIN 3 lesions (lower left photomicrograph) were purified from two affected members of Family X. The pancreatic cancer epithelial cells (lower right photomicrograph) were purified from a sample of sporadic adenocarcinoma.

### The 90 kDa *Palladin* Is the Major Isoform Expressed in Human Pancreatic Epithelium

At least three major isoforms of palladin are known, with molecular weights of 200 kDa, 140 kDa, and 90 kDa. The smallest isoform (90 kDa) is a component of the larger isoforms.

We used Western blotting and a polyclonal antibody (ab 621) to assess the protein isoform expressed in pancreatic ductal epithelium. The 90 kDa isoform is the predominant form of palladin in pancreatic ductal epithelium and in pancreatic cancer cell lines (Figure 7).

**Figure 7:**
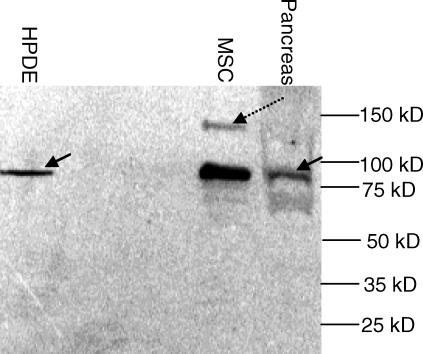
The 90 kDa Palladin Is the Major Isoform Expressed in Human Pancreatic Epithelium Proteins were detected using polyclonal antibody to palladin raised in rabbit (ab 621) in this Western blot. The major palladin isoform expressed in normal HPDE and cultured epithelial cells is the 90 kDa isoform (solid arrows). The positive control sample (MSC, human mesenchymal stem cells) also has the major 90 kDa isoform, but the 140 kDa isoform is also present (dashed arrow). Mesenchymal stem cells express high levels of palladin.

### *Palladin* and Alpha-Actinin Proteins Are Abnormally Expressed in Sporadic Pancreatic Cancer Cell Lines

It has been shown that the 90 kDa palladin isoform colocalizes in stress fibers with several cytoskeletal components, such as actin and alpha-actinin [16,19,25]. We examined the protein expression of these cytoskeletal components in pancreatic cancer cell lines using Western blotting and a polyclonal antibody (ab 621) to palladin. Of seven sporadic pancreatic cancer cell lines (FA6, HPAF, IMIMPC2, SUIT2, HS766T, PANC-1, and PATU2), five showed clear protein overexpression of palladin. Cell lines FA6, HPAF, IMIMPC2, SUIT2, and PATU2 overexpress palladin protein compared to the minimal expression in normal HPDE; alpha-actinin was also abnormally expressed in some of the pancreatic cancer cell lines, especially in PANC-1, a line that did not show overexpression changes in palladin protein (Figure 8). These data suggest that abnormal expression of palladin or its binding partner, alpha-actinin, is present in six of the seven pancreatic cancer cell lines we tested.

**Figure 8:**
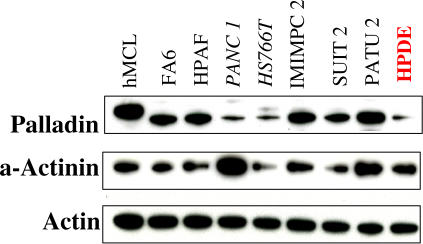
Abnormal Protein Expression of Palladin and Alpha-Actinin Proteins in Sporadic Pancreatic Cancer Cell Lines Human mesenchymal cell lysate (hMCL) was used as a positive control for palladin overexpression. FA6, HPAF, IMIMPC2, SUIT2, and PATU2 are sporadic pancreatic cancer cell lines that overexpress palladin protein compared to the minimal expression evident in normal HPDE. Alpha-actinin is also abnormally expressed in some of the pancreatic cancer cell lines compared to normal pancreas—especially in PANC-1, one of the few pancreatic cancer lines that does not show expression changes in palladin protein.

### Mutations in Sporadic Pancreatic Cancer Cell Lines

To evaluate the etiology of palladin overexpression in sporadic pancreatic cancer, we sequenced the DNA of the seven sporadic pancreatic cancer cell lines. We identified a separate point mutation in a conserved coding region of *palladin* present in one of the sporadic pancreatic cancer cell lines (unpublished data). The new and second mutation identified in sporadic pancreatic cancer supports the broader role of *palladin* as a proto-oncogene in sporadic pancreatic cancer.

### Expression of the Mutant Palladin Construct Results in an Abnormal Cellular Cytoskeleton

To investigate if the mutant form of palladin causes abnormalities in cytoskeletal formation, we cloned the coding region for the normal human 90 kDa isoform sequence into a vector with a GFP tag upstream, designated as WT construct. We then used site-directed mutagenesis to create a C to T mutation at cDNA position 715 bp, matching the Family X mutation, designated as FX construct. These constructs were transfected into HeLa cells to study the phenotypes. Cells transfected with the wild-type *palladin* construct displayed well-organized filaments that colocalized with the F-actin bundles (Figure 9A and 9B). When HeLa cells were transfected with the FX construct, however, the GFP-mutant palladin forms atypical cytoskeletal aggregates. Perhaps the most distinctive phenotype for the FX construct transfection was the presence of multiple small flower-like structures in the cytoplasm: the center of the flower colocalized with F-actin, while the green flower “petals” of mutated palladin did not colocalize with F-actin (Figure 9C and 9D). Other cells that express the mutated palladin contained flecks of protein in the cytoplasm of the cells that did not colocalize with F-actin (Figure 9E and 9F). These changes occurred at a much higher frequency in FX than in WT controls. Cells transfected with the control GFP empty vector exhibited a green haze throughout the whole cells (unpublished data). Taken together, these results suggest that mutant palladin may lose its ability to fully participate in the regulation of actin networks, impairing the normal organization of the cytoskeleton.

**Figure 9:**
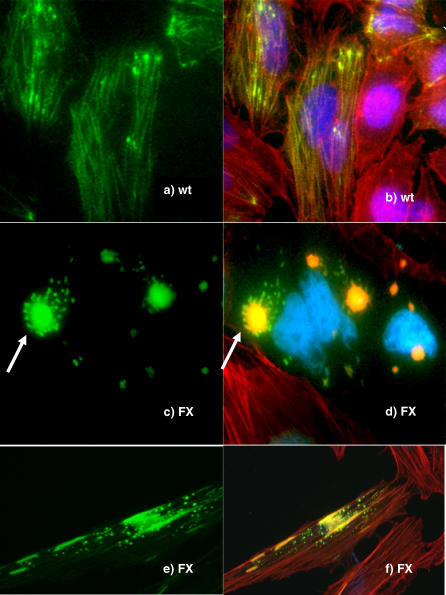
Expression of Human Wild-Type and P239S Mutant *Palladin* Constructs in HeLa Cells (A and B) When HeLa cells were transfected with the human wild-type (WT) *palladin* construct, filamentous bundles (A) clearly colocalized with the F-actin (B). (C and D) In contrast, expression of the mutant P239S Family X (FX) construct resulted in large palladin flower-shaped structures (arrows) in which the center, but not the petals of the flower, colocalizes with actin. (E and F) Expression of Family X constructs can also cause small cytoplasmic flecks of palladin that do not colocalize with actin. HeLa cells overexpressing the empty GFP vector displayed uniform distribution of GFP in the whole cells that did not colocalize with F-actin (unpublished data). Green, GFP-palladin; red, F-actin; blue, nucleus; yellow, colocalized palladin and F-actin. All figures are at 100× magnification.

### Mobility Is Increased in Cells that Express Mutant Palladin

We hypothesized that the cytoskeletal changes in the cells containing the mutated Family X construct might provide a cancer phenotype, specifically increased cell motility. The cytoskeleton is essential for cell movement, and cell mobility is important for the invasive nature of cancer cells. HeLa cells transfected with one of three constructs of *palladin* (FX, WT construct, or an empty vector) were individually plated onto a fibronectin-coated membrane in a transwell chamber, and a migration assay was performed in a standard fashion [26]. The cells transfected with the FX construct encoding P239S mutant palladin outpaced the other cells at every time point. On average, 33% more cells with the FX construct migrated through the transwell than the cells with WT construct and 40% more than the cells with the empty vector (Figure 10). Therefore, P239S palladin expression induces increased cell motility, consistent with a proposed oncogenic function.

**Figure 10:**
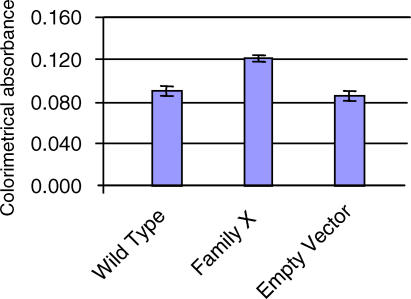
Migration Assay of *Palladin* Constructs in HeLa Cells The cells expressing the Family X *palladin* construct (P239S) migrated fastest—33% and 42% faster than the cells expressing wild-type (WT) protein and empty vector constructs, respectively.

## Discussion

The data presented here identify *palladin* as the mutated gene in the pancreatic cancer susceptibility locus at 4q32–34, validate abnormal expression of the gene in a familial pancreatic cancer kindred, demonstrate RNA overexpression of this gene in sporadic pancreatic cancers and precancerous pancreatic tissues, characterize the functional changes induced by the mutant protein, and suggest that cytoskeletal abnormalities may be a driving force in pancreatic oncogenesis.

*Palladin* is named for a Renaissance architect, Palladio, because of its role as a key architectural element of the cell. The function of palladin is to provide a scaffold for cytoskeletal proteins to bind, forming the actin filament complexes necessary for cell morphology, movement, and differentiation [16]. Abnormalities in the proteins that arrange the cytoskeleton can result in loss of cellular polarity (as the cytoskeleton helps direct the location and size of the nucleus in the cell), changes in cell size, increased invasiveness (advancement and retraction of the lamellipodia moving the cell), and abnormal signaling within the cell (the cytoskeleton scaffold is the docking point for many protein-protein interactions).

The cytoskeleton is composed of actin bundles (polymerized filaments) that form the “tent poles” supporting the cell membrane. The tented cell is held onto the basement membrane by integrin pegs, with the cytoskeleton directly connected to the pegs. It is through this normal integrin-cytoskeleton interaction that the cell is able to distinguish down from up, providing normal polarity for the cell and a stationary position [27]. During cell migration, the cross-linked network of actin filaments is dynamic, forming and dissolving through regulation by specialized actin-binding proteins; in essence these actin-binding proteins form complexes and a scaffold for the actin to polymerize. Disruption of the cytoskeleton has been directly linked to metastatic potential of cancer cells [28]. Specifically, highly metastatic cells, which have poor cytoskeletal architecture, can detach easily from the primary tumor mass, form transient and weak connections with surrounding connective tissue, and rapidly migrate through it [29].

To make the discovery of mutated *palladin* as a cause of familial pancreatic cancer possible, we had to devise an endoscopic surveillance program that could detect the precancerous lesions of the pancreas. This program, to our knowledge the first of its kind, allowed the identification of affected members in Family X, a large kindred with autosomal dominant, highly penetrant pancreatic cancer. Genotyping linked the pancreatic susceptibility gene to 4q32–34. A custom microarray of the dense 4q32–34 region led to the detection of *palladin* overexpression in Family X precancer and in sporadic cancer as well. RNA overexpression of *palladin* was validated by qRT-PCR in whole tissue of four precancerous samples (two from members of Family X and two from non-Family X patients) and in 16 sporadic cancers. *Palladin* was also overexpressed in the apparently “normal” tissue adjacent to the cancer. Specific analysis of the ductal epithelium revealed an apparent dose-response of *palladin* levels in the pancreatic epithelial cells, with increasing RNA expression directly associated with increasing neoplastic degeneration. These findings suggest that *palladin* plays a very early role in pancreatic tumorigenesis in both the familial and sporadic forms of the disease and that malignant progression parallels increased expression of the gene.

Once the candidate gene was identified, sequencing verified that a mutation in the cytoskeleton scaffold gene *palladin* causes familial pancreatic cancer in Family X. We demonstrated that the mutated allele is expressed and that the consequence of the P239S mutation is a change in amino acids at the essential alpha-actinin binding site leading to distinctive changes in the actin bundle morphology. Cells expressing the P239S mutant palladin protein were found to be significantly more mobile than those expressing wild-type palladin—theoretically, this capability would provide an advantage for cells to invade surrounding structures (a signature feature of cancer).

Palladin is a key scaffold for the cytoskeleton—it binds several proteins including alpha-actinin [20]. The *palladin* mutation in Family X leads to an amino-acid change in the highly conserved and essential binding site for alpha-actinin (Figure 5) [20]. We were able to detect protein expression abnormalities in either palladin and/or alpha-actinin in six of seven of the sporadic pancreatic cancer cell lines that we tested. This change in the palladin/alpha-actinin axis suggests that these proteins are essential for intact normal cellular behavior. Specifically, the alpha-actinin/palladin protein axis establishes a link between B-integrin subunits and filamentous actin, holding the cell (through the cytoskeleton) to the basement membrane (unpublished data) [16,27]. Through this action, normal pancreatic epithelial cells are uniform in size, oriented to the basement membrane, and immobile—exactly the cellular features that are lost in cancer. Alpha-actinin also plays a role in formation of complex/adhesion plaques; these plaques are modulated during growth and differentiation (and reduced in tumor cells) [29]. Mutations in the alpha-actinin binding site of palladin have not previously been recognized in human disease, although decreased levels of normal alpha-actinin in 3T3 cells cause tumorigenesis in nude mice [30].

Complete loss of palladin function in knock-out mice results in embryonic lethality [31]. It is apparent that at least some palladin protein must be present for the cell differentiation and migration events that occur during normal development. Mutation, rather than complete loss, may lead to partially intact but imperfect cytoskeleton scaffold function. In support of this hypothesis, the Family X mutated constructs revealed that actin cytoskeleton bundles can still form, although they are aberrant.

It appears likely that *palladin* is a proto-oncogene. The protein is overexpressed in sporadic pancreatic cancer, and even in the normal-appearing tissue adjacent to the cancer. The molecular events that cause this overexpression are unknown. Copy number in the *palladin* region (4q32–34) has not been reported to be abnormal (either gains or losses) in previous comparative genomic hybridization analyses [22]. The copy number analysis that we performed in the precancerous tissue of Family X members revealed two copies of the gene in every sample tested. These findings make it less likely that palladin overexpression is achieved through amplification. Rather, palladin levels might be elevated through point mutations, such as the one in Family X, or through other mechanisms, such as promoter region changes, epigenetic changes, or regulatory factor alterations. It is also likely that palladin does not act alone. The vast majority of pancreatic cancers have activation of the *K-ras* oncogene, including in Family X [7]. The idea that *palladin* and *K-ras* may work together is an interesting conjecture. Both of the encoded proteins regulate normal cytoskeletal activity, and the joint overexpression and activation of these genes might induce inescapable failure of normal cytoskeleton function.

Pancreatic cancer biology has been difficult to probe. Although genetic defects associated with pancreatic cancer have been described, a cohesive explanation of pancreatic neoplastic progression is lacking. The concept that the cytoskeleton could play a key role in pancreatic cancer formation is compelling. The critical features that underlie cancer, including nuclear atypia, loss of cellular polarity, altered cell morphology, increased mobility, and invasion into surrounding structures, are all linked to the dynamic behavior of the cytoskeleton. Moreover, altered cytoskeletal tension can distort the attached nuclear envelope, rearranging chromatin/gene positions, subjecting DNA to increased mutations, and inducing resistance to chemotherapeutic agents [32–34].

Our data support a key role for *palladin* in the formation of sporadic pancreatic cancer and precancer; moreover an inherited mutation in the *palladin* gene leads to a highly penetrant autosomal dominant form of familial pancreatic cancer. However, the work described is limited to one familial pancreatic cancer kindred—it will be interesting to evaluate additional familial pancreatic cancer kindreds for mutational changes in palladin. The work is also limited in terms of understanding the role of palladin in sporadic pancreatic cancer. It seems unlikely that the P239S germline mutation will be a useful tool in genetic testing for pancreatic cancer risk in individuals without a strong family history of pancreatic cancer.

Many questions remain. Do somatic *palladin* mutations play a role in pancreatic cancer? How is the gene activated in the sporadic setting? Are other binding partners of palladin abnormally expressed or mutated in pancreatic cancer (sporadic or familial)? How does palladin interact with the products of oncogenes and tumor suppressor genes in known pancreatic cancer pathways? Answers to these questions are crucial to improved methods of early detection and develop new treatments for this devastating disease.

## Abbreviations

FX: Family X mutant construct
GFP: green fluorescent protein
HPDE: human pancreatic ductal epithelium
qRT-PCR: quantitative RT-PCR
WT: wild type
